# Osteoporosis-related life habits and knowledge about osteoporosis among women in El Salvador: A cross-sectional study

**DOI:** 10.1186/1471-2474-5-29

**Published:** 2004-08-26

**Authors:** Roberto Hernandez-Rauda, Sandra Martinez-Garcia

**Affiliations:** 1 Facultad de Ciencias de la Salud, Universidad Andrés Bello, 1^a ^Calle Poniente y 41^a ^Avenida Norte, 2128, Colonia Flor Blanca, San Salvador, El Salvador, América Central

## Abstract

**Background:**

Osteoporosis is a systemic skeletal disorder, characterized by reduced bone mass, deterioration of bone structure, increased bone fragility, and increased fracture risk. It is more frequent to find among women than men at a 4:1 ratio. Evidence suggests that to adopt changes on some life habits can prevent or delay development of osteoporosis. Several osteoporosis-risk factors have been confirmed in the US and western Europe, but in El Salvador there are neither reliable epidemiological statistics about this skeletal disorder nor studies addressing osteoporosis-risk factors in women. The aim of this study was to determinate the extent of osteoporosis knowledge, the levels of both daily calcium intake and weight-bearing physical activity, and the influence of several osteoporosis-risk factors on these variables in three age groups of Salvadorean women.

**Methods:**

In this exploratory cross-sectional study, an osteoporosis knowledge assessment questionnaire incluiding a food frequency and a physical activity record section were used to collect data and it was delivered through a face-to-face interview. A convenience sample (n = 197) comprised of three groups of women aged 25–35 years, 36–49 years, and over 49 years was taken. Among-group comparisons of means were analyzed by two-way ANOVA. To determinate the overall influence of osteoporosis-risk factors, the multivariate analysis was used.

**Results:**

Study results indicated that better educated women had more knowledge about osteoporosis than women with a low education level, regardless of age, even though this knowledge was rather fair. Older women got more weight-bearing physical activity at home and less at place of employment than reported by the younger women; however, neither group performed sufficient high-intensity WBPA to improve bone mass. Regardless of age, the most women consumed 60% or less than the Dietary Reference Intake of calcium and depend on household income, lactose intolerance and coffee rather than milk consumption.

**Conclusion:**

In summary, the majority of women in this study have modest knowledge on osteoporosis. The knowledge base is not linked to preventive health habits, including sufficient calcium intake and performance of weight-bearing physical activities. They are thus at increased risk for low bone mass.

## Background

Osteoporosis is a systemic skeletal disorder, characterized by reduction of bone mass, deterioration of bone structure, increasing bone fragility, and increasing fracture risk [1-4]. It is more frequent among women than among men [5,6]. The development of low bone mass is typically asymptomatic, with many women reporting clinical manifestations including acute back pain, limited back mobility, fragility fractures (hip, vertebrae, proximal femur, distal radius, humerus, tibia), compression of midthoracic vertebrae and upper lumbar vertebrae, progressive deformation of the spinal column (cyphosis), reduced height, and radiculopathies [2,4-6,8,9].

Several risk factors for osteoporosis have been identified, these include female sex; Caucasian or Asiatic race; advancing age; family history of osteoporosis or fragility fractures; a low body mass index; menopause before age 45 years; prolonged amenorrhea unrelated to menopause; nulliparity; prolonged lactation; diet low in calcium and vitamin D; poor intestinal absorption of calcium; lactose intolerance; excessive caffeine or alcohol consumption; smoking; sedentary lifestyle; and prolonged treatment with thyroid hormones, glucocorticoids (e.g. cortisone), anticonvulsants, aluminum antiacids, and use of anticoagulants [1,3-6,8].

Approximately 20% of bone mass is genetically determined; however, the risk of osteoporosis can be reduced by optimizing bone mass increasing during youth, conserving bone mass during adulthood, and minimizing bone mass loss during advancing age [5-7]. Among most important preventive habits are a) weight-bearing exercise (e.g. going up and down stairs, jogging, aerobics, swimming, and isometrics; at least 30 minutes daily), b) diet or supplements containing adequate levels of calcium and vitamin D, and c) absence or cessation of smoking and no greater than moderate alcohol and/or caffeine consumption [5-10].

A study on US women aged over 25 years found that knowledge about osteoporosis was limited, irrespective of age [11]. Calcium intake was sufficient in most cases, but the amount and type of physical activity was inadequate to achieve enhanced bone mass; most women in this study performed some physical activity in the course of paid work or housework, but did not admit to systematic daily exercise. Other study on Caucasian and African-American women found that most of them had heard about osteoporosis, but few women got both adequate exercise and the recommended intake of calcium per day [12]. Asian women in Australia also had lower calcium intake (< 800 mg/day) and their knowledge about osteoporosis was limited [13].

There have been three related studies reported in Hispanic women: a study performed in Mexico [14], and two studies on women of Hispanic origin in the US [15,16]. The Mexican study considered women aged 50 – 59 years, and focused on knowledge about menopause and risk associated with premature menopause. About 90% of subjects were aware of the relationship between menopause and osteoporosis, but most subjects had little knowledge of other risk factors, and incorporated life habits that clearly increased osteoporosis risk. The studies on women of Hispanic origin in the US have yielded somewhat contradictory results. The first of these found that more than 37% of women had adequate preventive habits including the taking of calcium supplements and the performance of regular exercise; much of this was attributed to prior health education, knowledge about osteoporosis, bone-mass evaluation offered by healthcare services, and medical advice [15]. The second study considered both Hispanic and African-American women, and found that most women in both groups had a poor knowledge of behaviors that promote and maintain bone mass [16]. Notably, less than 50% of women performed regular physical exercise, and less than 10% had adequate calcium intake.

At present, the Salvadorean Public Health and Social Assistance Ministry does not maintain a specific record of this disease in adults, and most cases are likely classified as dorsalgy, that includes several musculoskeletal diseases such as radiculopathies, cervicalgy, lumbalgy, sciatica, spinal derived pain, and unspecified back pain [17]. This reflects the fact that osteoporosis may be reported as dorsalgy. According to this report from El Salvador, in 2001 dorsalgy was the seventh most frequent cause of morbidity in women aged 50 – 59 years attending outpatient clinics, with an incidence of 3,983 new cases per 100,000 inhabitants and a total of 8,989 consultations (first-time or subsequent); in women aged 60 years or more, there were 9,754 consultations for dorsalgy in 2001.

Bearing in mind the lack of reliable epidemiological data, the present study wished to investigate osteoporosis-related life habits (including exercise and calcium intake) and knowledge about osteoporosis among Salvadorean women aged ≥ 25 years. We investigated possible relationships of these variables with age, educational level, household income, family history of osteoporosis, menopause before age 45 years, fecundity (children per woman), lactose intolerance, caffeine consumption, low in calcium diet, and use of aluminum antiacids.

## Methods

### Study design and sampling

This was an exploratory study with a cross-sectional design performed between May and September 2003, and it was used to survey a convenience sample comprised of 197 women (73 aged 25 – 35 years, 74 aged 36 – 49 years, and 50 aged over 49 years) from urban areas within 6 main municipalities in El Salvador. All subjects were randomly sampled and recruited by personal contact through to visit homes, churches, schools, primary healthcare centers, hospitals, supermarkets, shopping centers, and parks. It was not necessary to obtain any proper informed consent from interviewed women. The participation rate of women was 87.5% (197 out of 225).

We selected three age groups of women attending following criterions: women aged 25 – 35 years are at the period when higher peak of bone mass is reached [3]; women aged 36 – 49 years are at a period around menopause when bones undergo a slow mineral density loss [3]; finally, most women aged over 49 years have undergone menopause, when osteoporosis clinical manifestations may begin to show [3].

### Data collection and instruments

Data were obtained at the time of interview, which were performed by a member of the research team. Prior training in interview techniques was obtained for study. Each staff interviewer subsequently participated in several practice sessions, with an evaluation component to confirm accurate transcription of responses, and comparison and adjustment to ensure good inter-interviewer concordance.

The data collection instrument incorporated a personal interview guide comprising five sections, validated through a prior pilot study performed in 12 women (4 in each age group).

Among the demographic information was household income. It was classified by the criteria of the Salvadorean Economy Ministry as "below poverty line" if monthly income was below the cost of two basic shopping baskets (i.e. < $254), and otherwise as "above poverty line" [18].

The second survey section generated information on family history of osteoporosis, and asked about the use of aluminum antiacids.

Section 3 addressed weight-bearing physical activity (e.g. walking, standing, climbing and descending stairs) both at place of employment and at home, and included questions on weight-bearing exercises including jogging, swimming, aerobics, and isometrics. This survey section was adopted from a previous study [11]. A test/ re-test procedure was administered a week apart on pilot study subjects to calculate reliability of next questionnaire sections. Test/re-test correlation was 0.67 for physical activity section.

Section 4 produced information on diet: for each of a list of 31 dietary and non-dietary items (including dairy products, baked products, meat, vegetables, fruits, and calcium supplements), the subjects were asked to estimate frequency of consumption (daily, weekly, fortnightly, or monthly). Similar checklists have been used in related previous studies [19]. Given the high coffee consumption in Central America, the subjects were asked about their degree of coffee consumption. Test/re-test correlation was 0.72 for dietary and non dietary calcium intake section.

Final section (5) comprised eight open questions designed to assess nine knowledge dimensions about osteoporosis in subjects. The nine knowledge dimensions regarding osteoporosis, their specific questions and corresponding scores (from 0 to 42) are shown in Table 1. These questions served as a backbone for the interview and they were similar to other set used in a previous study [11]. Test/re-test correlation was 0.59 for osteoporosis knowledge questionnaire section.

### Data analysis

**Figure 1:**
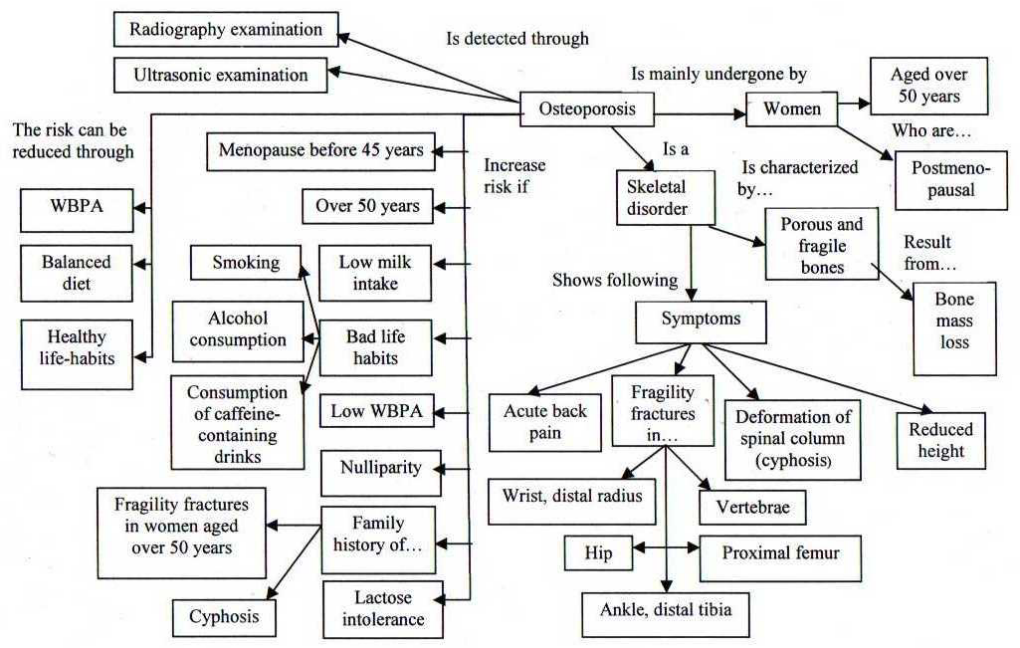
**Semantic map showing an osteoporosis knowledge structure. **Semantic map showing an osteoporosis knowledge structure related to several aspect concerning to concept, risk-factors, prevention, and diagnosis of this skeletal disorder.

Each subject's responses to the questions in the fifth section were analyzed by the principal researcher, who compared the answers with a semantic map (Fig. 1) developed by the authors on the basis of findings from previous studies on osteoporosis [1,2,5-7]. This approach allowed for quantification on knowledge about osteoporosis so: 5 points assigned for responses denoting knowledge of three or more concepts (each node in semantic map represents a concept), 3 points for responses denoting knowledge of 2 concepts, and 2 points for responses denoting knowledge about one out of osteoporosis-related concepts as reported previously [11]; except for osteoporosis information source dimension since its sub-score depended on number of information sources that women were able to mention, so that they only got one point per each source up to a maximum of 5 points.

The total score achievable was 42 points. The total number of hours of weight-bearing physical activity was estimated by adding the subtotals for activities at home, activities at worksite, and that of weight-bearing exercises.

Total calcium intake was estimated by adding the estimated subtotals for consumed dairy products, other dietary intake, and calcium supplements, including calcium content per portion × the number of portions per day. Obtained values were compared with reference values [20].

Data are cited in the text as means ± standard errors. For variables with homogeneous variance, means were compared by two-way analysis of variance and by Tukey tests for pairwise comparisons. For variables with non-homogenous variance, data were compared by the Kruskal-Wallis test (ANOVA on ranks) followed by Dunn's test for pairwise comparisons. These analyses were performed using SigmaStat version 2.03. Correlation analysis was used to determine relationships between knowledge scores and WBPA including exercise and calcium intake. To develop predictive models, we used a multivariate analysis based on multiple linear regression as contained in the program AMOS version 5 (Small Waters Corp.).

## Results

### Characteristics of the interviewed women

The participant characteristics of each age group are shown in Table 2. Frequency of women with none or only a primary education increased with age; conversely, frequency of women with secondary or higher education decreased with age. Household income reported that most women (> 61%) were below the poverty line; although the number of women living on poverty conditions fluctuated with age. The frequency of nulliparity among interviewed women declined with age, whereas both the frequency of parity among interviewed women and the fecundity rate (children per woman) increased with age as anticipated.

In all age groups, more than 44% of interviewed women reported a family history of osteoporosis, which included extended family: grandmothers, mothers, aunts, elder sisters, and cousins. Median, mean (SEM), and range of age of menopause in interviewed women were: 44, 42.3 (± 0.8), and 27–53 years, respectively. About 20% of interviewed women had lactose intolerance or ingested aluminum antiacids, both of these characteristics were unrelated of age. In addition, most women (> 69%) were coffee-consumers and the consumption of this drink increased with age.

### Knowledge about osteoporosis

The sub-scores per knowledge dimensions about osteoporosis and age groups are shown in Table 3. The total scores of knowledge regarding osteoporosis were similar in the three age groups of women (median 14 in 25- to- 35-years; 17 in 36- to 49-years; 15 in over 49 years).

Most of interviewed women (75%) had enough knowledge about osteoporosis regarding the concept of disorder and its risk factors, sex-related factor, and prevention behaviours, irrespective of age (Table 3). Conversely, these women got less knowledge scores for diagnosis and treatment of osteoporosis than other examined dimensions.

**Figure 2:**
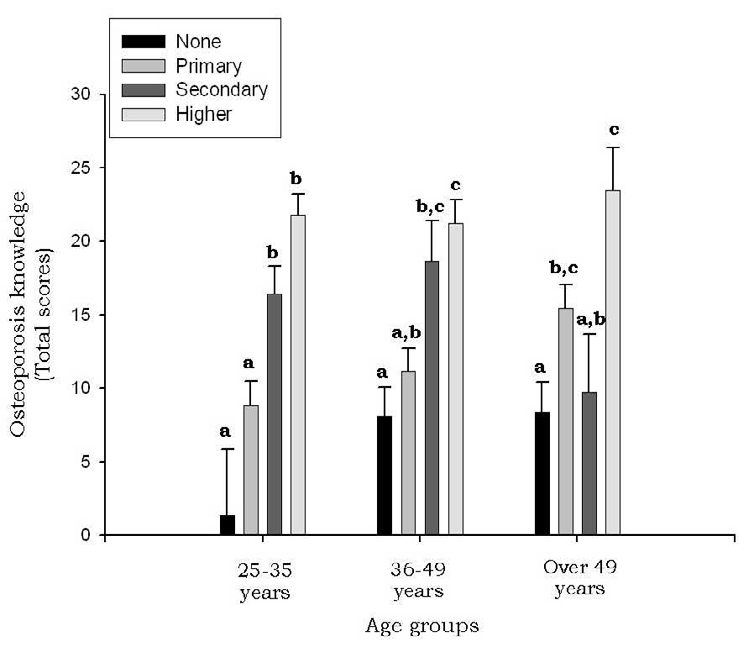
**Total osteoporosis knowledge scores. **Total osteoporosis knowledge scores obtained by Salvadorean women in each age group (25 – 35 years, n = 73; 36 – 49 years, n = 74; over 49 years, n = 50), subclassified by educational level (none, primary, secondary, higher). Data are means ± SEM. Bars with distinct letters differ significantly within each age group (p < 0.05; two-way ANOVA followed by Tukey test).

In all three age groups, women with secondary or higher education obtained significantly higher total knowledge scores (*F *= 22.46, *p *< 0.001) than women with lower educational level (Fig. 2). There was not a significant relationship between age and educational level (*F *= 1.38, *p *= 0.223). Only 5% of the women with higher education obtained total scores of less than 12 points, and 75% of them obtained total scores of more than 25 (table 3).

**Figure 3:**
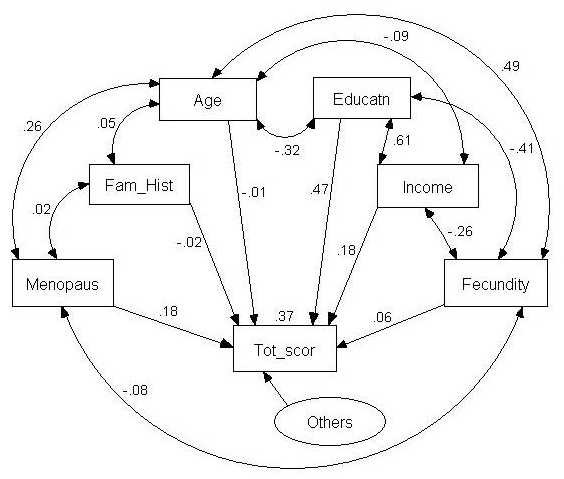
**Predictive model of influences of surveyed variables on total knowledge scores. **Predictive model of the surveyed variables: age (Age), education (Educatn), family history of osteoporosis (Fam_Hist), household income (Income), menopause (Menopaus), and fecundity (Fecundity) influencing on total osteoporosis knowledge score (Tot_scor; R^2 ^= 0.37, p < 0.001, n = 197). The numbers on single- or double-headed arrows are regression weights or correlation coefficients, respectively.

Multivariate analysis (factors age, educational level, family history of osteoporosis, household income, early menopause, fecundity) explained only about 37% of variance in total score (R^2 ^= 0.367, p < 0.001) (Fig. 3). The most meaningful predictors of total score were educational level (R = 0.47, p < 0.001), household income (R = 0.18, p = 0.015), and early menopause (R = 0.18, p = 0.003).

### Physical activities

The amount of weight-bearing physical activity was similar in the three age groups (mean 8.7 ± 0.5 hours/day in women 25- to 35 years; 8.9 ± 0.5 hours/day in those 36- to 49 years; 8.3 ± 0.6 hours/day in those over 49 years). Analysis of variance with factors of age and family history of osteoporosis indicated that neither factor produced a significant effect (F = 0.23, p = 0.794; F = 0.04, p = 0.852), nor the interaction between them was significant.

**Figure 4:**
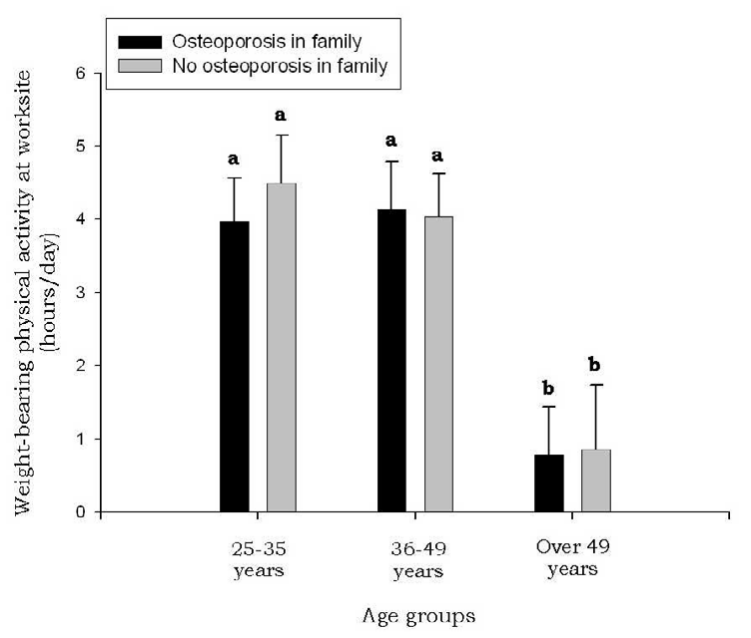
**Weight-bearing physical activities performed at worksite. **Weight-bearing physical activity (hours/day) at worksite performed by Salvadorean women in each age group (25 – 35 years, n = 73; 36 – 49 years, n = 74; over 49 years, n = 50), subclassified by family history of osteoporosis (osteoporosis in family or no osteoporosis in family). Data are means ± SEM. Bars with distinct letters differ significantly (p < 0.05; Kruskal-Wallis' test followed by Dunn's test).

**Figure 5:**
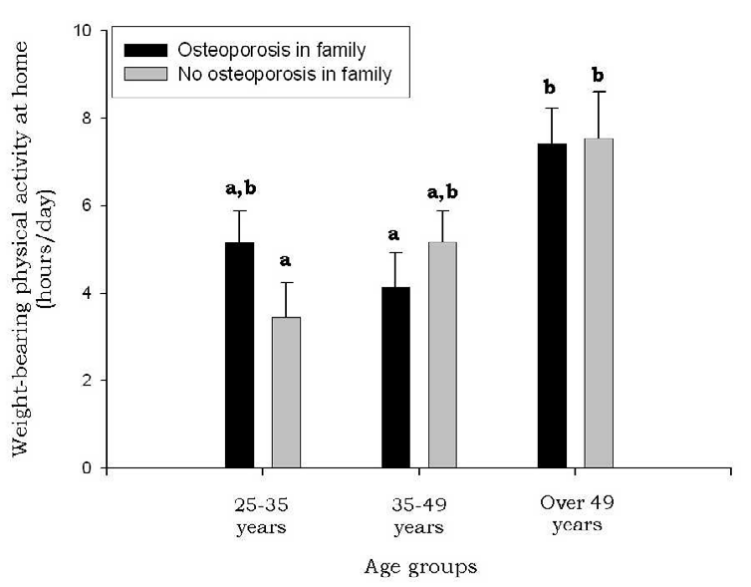
**Weight-bearing physical activities performed at home. **Weight-bearing physical activity (hours/day) at home performed by Salvadorean women in each age group (25 – 35 years, n = 73; 36 – 49 years, n = 74; over 49 years, n = 50), subclassified by family history of osteoporosis (osteoporosis in family or no osteoporosis in family). Data are means ± SEM. Bars with distinct letters differ significantly (p < 0.05; Kruskal-Wallis' test followed by Dunn's test).

**Figure 6:**
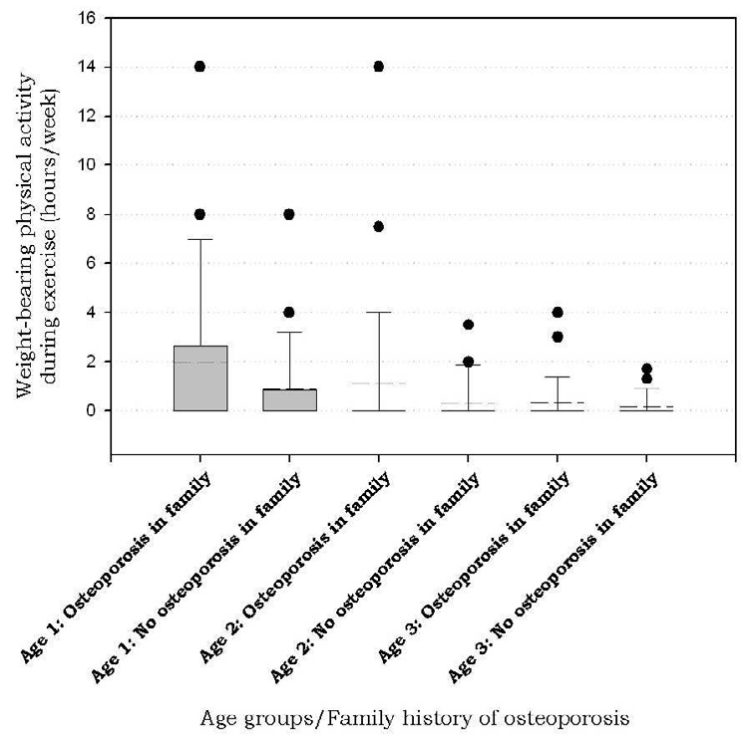
**Weight-bearing physical activity during exercise. **Weight-bearing physical activity during exercise (hours/week) performed by Salvadorean women in each age group (Age 1: 25 – 35 years, n = 73; Age 2: 36 – 49 years, n = 74; Age 3: over 49 years, n = 50), subclassified by family history of osteoporosis (osteoporosis in family or no osteoporosis in family). Data are means ± SEM. Bars with distinct letters differ significantly (p < 0.05; Kruskal-Wallis' test followed by Dunn's test).

The amount of weight-bearing physical activity reported at the worksite was markedly and significantly lower in over 49 years than in the other two age groups (H = 33.15, *p *< 0.001; Fig. 4). Conversely, physical activity at home was higher in women over-49 years than reported in the other two age groups (H = 19.34, p = 0.002; Fig. 5). The amount of weight-bearing exercise was low in all three groups, with means about 2 hours per week in the 25- to 35-years, and less than 1 hour per week in the older age groups (Fig. 6). About 75% of 25- to 35 years appear to do less than 2.7 hours of weight-bearing exercises per week; while about 75% of women in the older age groups reported doing no exercises (Fig. 6). However, the total duration of exercise did not vary significantly among the three age groups, or between women with or without a family history of osteoporosis (*H *= 10.25, *p *= 0.069).

**Figure 7:**
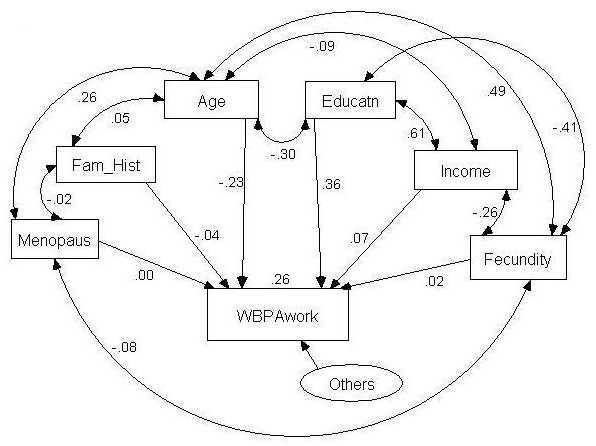
**Predictive model of influences of surveyed variables on WBPA performed at worksite. **Predictive model of the surveyed variables: age (Age), education (Educatn), family history of osteoporosis (Fam_Hist), household income (Income), menopause (Menopaus), and fecundity (Fecundity) influencing on amount of weight-bearing physical activities performed at worksite (WBPAwork; I; R^2 ^= 0.26, p < 0.001, n = 197). The numbers on single- or double-headed arrows are regression weights or correlation coefficients, respectively.

**Figure 8:**
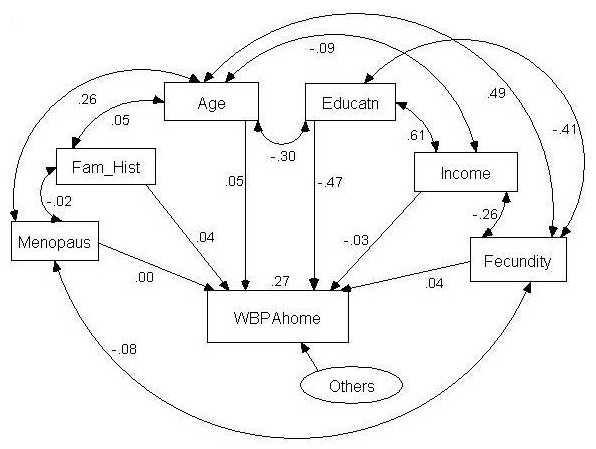
**Predictive model of influences of surveyed variables on WBPA performed at home. **Predictive model of the surveyed variables: age (Age), education (Educatn), family history of osteoporosis (Fam_Hist), household income (Income), menopause (Menopaus), and fecundity (Fecundity) influencing on amount of weight-bearing physical activities performed at home (WBPAhome; II; R^2 ^= 0.27, p < 0.001, n = 197). The numbers on single- or double-headed arrows are regression weights or correlation coefficients, respectively.

Multivariate analysis employing dependent variable of amount of total physical activity and candidate predictors age, educational level, household income, family history of osteoporosis, early menopause and fecundity, appeared to explain only 5% of total variance (R^2 ^= 0.048, p = 0.038). Use of dependent variable of physical activity at worksite explained about 26% of variance (R^2 ^= 0.264, p < 0.001; Fig. 7); the most effective predictors were educational level (R = 0.361, p < 0.01) and age (R = -0.234, p = 0.002). Similarly, use of dependent variable of physical activity at home explained about 27% of variance (R^2 ^= 0.273, p < 0.001; Fig. 8); again the most effective predictor was age (R = -0.471, p < 0.001). Use of dependent variable of exercise activity explained only 13% of variance (R^2 ^= 0.128, p < 0.001); the most effective predictor was family history of osteoporosis (R = 0.169, p = 0.011).

Total osteoporosis knowledge score was not predicted on the amount of weight-bearing exercise (R = 0.081, p = 0.218). Although, total osteoporosis knowledge scores were significantly associated with WBPA at worksite (R^2 ^= 0.07, p < 0.001), at home (R^2 ^= 0.05, p = 0.002) or during exercise (R^2 ^= 0.04, p = 0.004), squared correlations were rather low in all of cases.

### Dietary calcium intake

**Figure 9:**
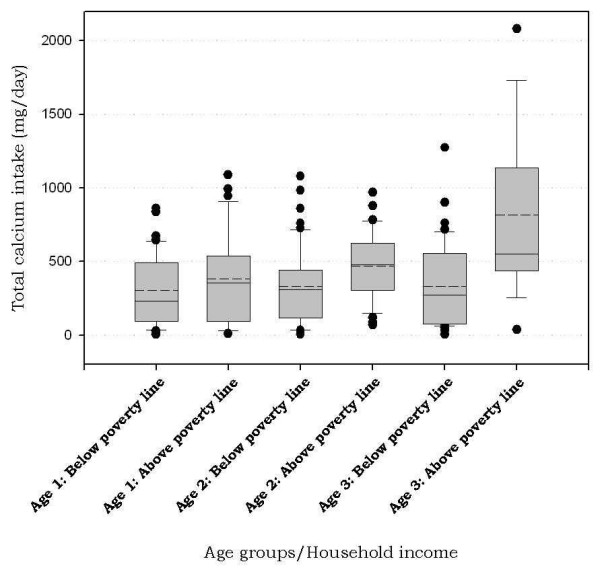
**Total calcium intakes among Salvadorean women classified by age and household income. **Total calcium intakes among Salvadorean women in each age group (Age 1: 25 – 35 years, n = 73; Age 2: 36 – 49 years, n = 74; Age 3: over 49 years, n = 50), subclassified by household income (above or below the poverty line). Shaded boxes extend from the 25^th ^to the 75^th ^percentile; whiskers indicate the 5^th ^and 95^th ^percentiles; dots indicate individuals lying outside these percentiles; the mean is indicated by a solid line, the median by a dashed line.

Figure 9 summarizes the data on total calcium intake (mg/day) in the three age groups, subdivided into women above and below the poverty line. Independently of income, about 75% of women aged less than 49 years ingest less than 600 mg/day of calcium (i.e. only about 60% of the recommended daily intake, 1000 mg/day). In the over-49 age group (recommended daily intake 1200 mg/day), there is a marked difference in calcium intake between women above and below the poverty line; with women below the poverty line typically showing very low calcium intake (over 75% ingest less than 600 mg/day), whereas calcium intake in women above the poverty line is higher (though nevertheless lower than the recommended daily intake in over 75% of subjects).

**Figure 10:**
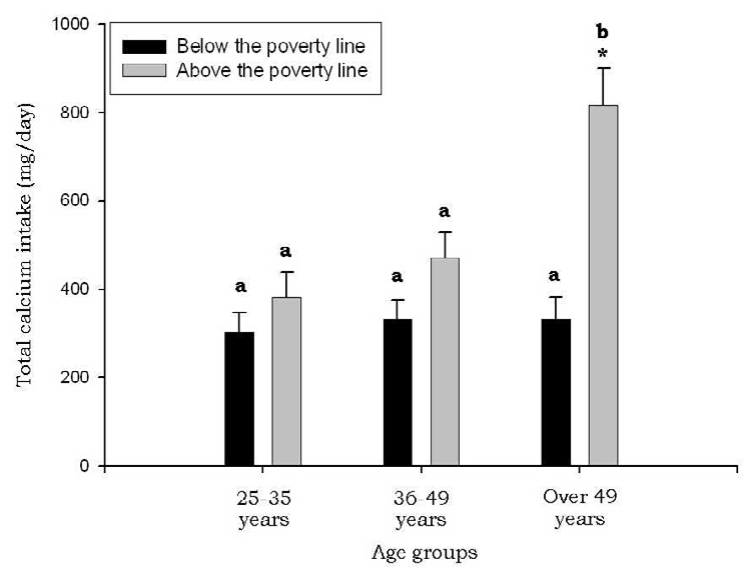
**Total calcium intake among Salvadorean women classified by age and household income. **Total calcium intake among Salvadorean women in each age group (25 – 35 years, n = 73; 36 – 49 years, n = 74; over 49 years, n = 50), subclassified by household income (above or below the poverty line). Data are means ± SEM. Bars with distinct letters differ significantly (p < 0.05; Kruskal-Wallis' test followed by Dunn's test).

**Figure 11:**
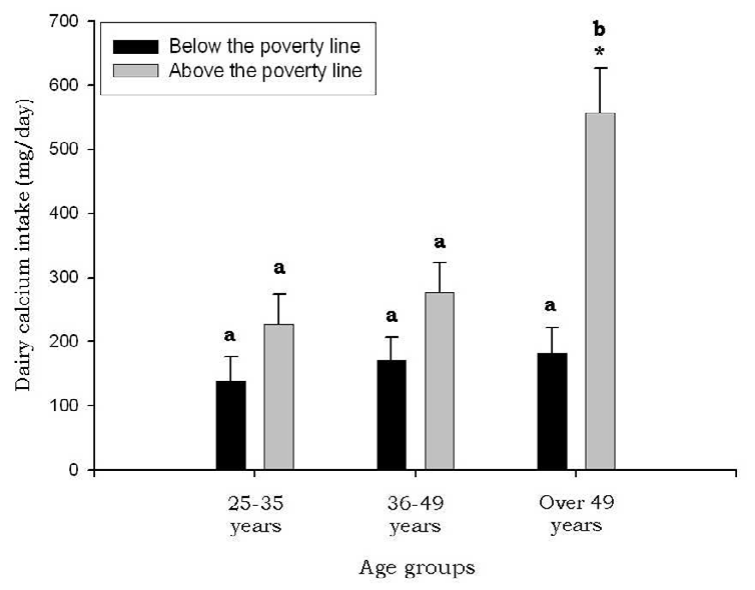
**Dairy calcium intake among Salvadorean women classified by age and household income. **Dairy calcium intake among Salvadorean women in each age group (25 – 35 years, n = 73; 36 – 49 years, n = 74; over 49 years, n = 50), subclassified by household income (above or below the poverty line). Data are means ± SEM. Bars with distinct letters differ significantly (p < 0.05; Kruskal-Wallis' test followed by Dunn's test).

Calcium intake was estimated through the use of dairy products, with 75% of women ingesting less than 410 mg/day, in all three age groups below the poverty line, and in the two younger age groups above the poverty line. In the over-49 above-poverty-line group, 75% of women ingest less than 775 mg/day. Dairy products contributed on average 57% of total calcium intake in women below the poverty line, versus 68% of total calcium intake in women above the poverty line. Women in the over-49 above-poverty-line group ingested significantly more total calcium (H = 18.36, p = 0.003) and significantly more dairy calcium (H = 18.97, p = 0.002) than women in all other age/income groups (Figs. 10, 11).

Supplementary calcium intake did not vary significantly among the age/income groups (H = 9.04, p = 0.108), neither was there any significant interaction between these factors.

**Figure 12:**
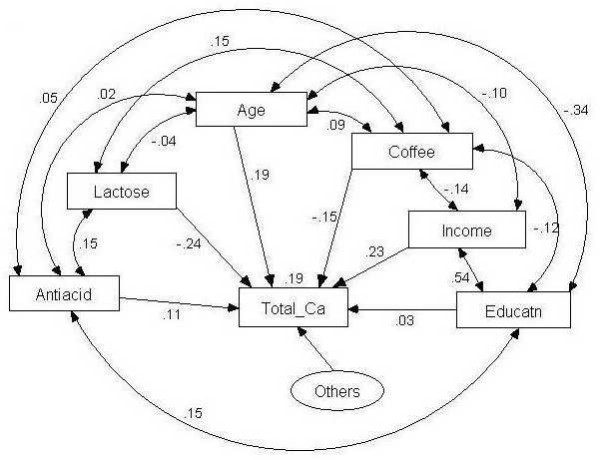
**Predictive model of influences of surveyed variables on total calcium intake. **Predictive model of the surveyed variables: age (Age), lactose intolerance (Lactose), coffee consumption (Coffee), use of aluminum antiacids (Antiacid), household income (Income), and education (Educatn) influencing on total calcium intake (Total_Ca; I; R^2 ^= 0.19, p < 0.001, n = 197). The numbers on single- or double-headed arrows are regression weights or correlation coefficients, respectively.

**Figure 13:**
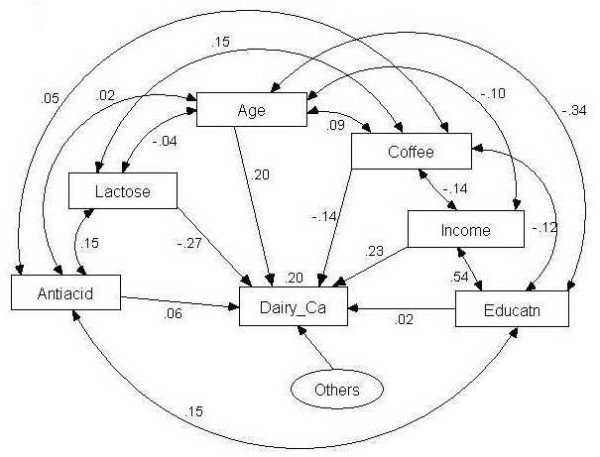
**Predictive model of influences of surveyed variables on dairy calcium intake. **Predictive model of the surveyed variables: age (Age), lactose intolerance (Lactose), coffee consumption (Coffee), use of aluminum antiacids (Antiacid), household income (Income), and education (Educatn) influencing on dairy calcium intake (Dairy_Ca; II; R^2 ^= 0.20, p < 0.001, n = 197). The numbers on single- or double-headed arrows are regression weights or correlation coefficients, respectively.

Multivariate analysis with dependent variable of total calcium intake and candidate predictors age, educational level, household income, lactose intolerance, coffee consumption and use of aluminum antiacids, provided a model that explained only 19% of total variance (R^2 ^= 0.191, p < 0.001) (Fig. 12). With the addition of dairy calcium intake, the model explained 20% of total variance (R^2 ^= 0.204, p < 0.001) (Fig. 13). In both cases, the most effective predictors were lactose intolerance (total calcium intake R = -0.239, p < 0.001; dairy calcium intake R = -0.272, p < 0.001), household income (R = 0.229, p = 0.003; R = 0.232, p = 0.002), age (R = 0.191, p = 0.005; R = 0.198, p = 0.004), and coffee consumption (R = -0.146, p = 0.025; R = -0.141, p = 0.029).

Women who had moderate osteoporosis knowledge had an increase in their intake of calcium that was significant (R = 0.142, p = 0.045). Although total osteoporosis knowledge scores were significantly associated with total calcium intake (R^2 ^= 0.06, p < 0.001) or dairy calcium intake (R^2 ^= 0.04, p = 0.008) or non-dairy calcium intake (R^2 ^= 0.03, p = 0.016) or calcium supplements (R^2 ^= 0.07, p < 0.001), squared correlations were rather low in all cases.

With the addition of calcium supplement intake only 12% of total variance was accounted for (R^2 ^= 0.119, p = 0.003). The greatest predictors were a woman's use of aluminum antiacids (R = 0.232, p < 0.001) and her educational level (R = 0.207, p = 0.004).

## Discussion

The total scores regarding nine knowledge osteoporosis dimensions were similar in all age groups of interviewed women (range of median 14 – 17, average 12.1 – 14.8) out of a possible 42 points. A similar study found Taiwanese women got a mean score of 15 out of 44 points related to six osteoporosis knowledge dimensions [21], whereas surveyed American women of three age groups got averaged knowledge scores from 32 to 44 points out of 183 [11]. In all cited cases, the obtained scores indicate that knowledge about osteoporosis is poor or limited among surveyed subjects so health educational programs and health services regarding osteoporosis are necessary for Salvadorean women, as it is also suggested for Taiwanese [21] and American women [11] of all ages.

The present results also indicate that Salvadorean women with secondary or higher education have significantly better knowledge of osteoporosis than women with a low educational level, regardless of age. Similarly, other study found that better educated Chinese women in Singapore seem to know more about osteoporosis than those ones worst educated [22]. A previous study has likewise found that osteoporosis-related knowledge is independent of age [11]. Our multivariate analyses indicated that the most effective predictors of osteoporosis-related knowledge were educational level, household income, and early menopause. This latter factor perhaps affects osteoporosis-related knowledge through to give brochures and magazines to women at shopping centers, supermarkets, physician's clinics, schools, and colleges by non governmental organizations such as Salvadorean Demographical Association, dairy good producers such as New Zealand Dairy Board and Australian Milk Products, and some pharmaceutical laboratories, especially those produce calcium supplements. As well as information presented on television and in the press.

Besides, short counselling sessions about preventive aspects of osteoporosis are given by some physician's private clinics, because there is not a settled public health education program about osteoporosis in El Salvador. This country is not only case in Latin America, since Mexican women got more osteoporosis information from mass communication media than health education activities of public institutions [14]. However, total knowledge about osteoporosis may not lead to an improvement in health lifestyle; it is necessary to know more about some specific aspects as osteoporosis risk factors and to acquire healthy habits to reduce the risk for low bone mass. In multivariate analyses, knowledge about osteoporosis was not a significant predictor of either amount of physical activity or total calcium intake. Previous studies likewise found that knowledge of osteoporosis does not correlate with risk-reducing life habits [11,12]. These authors suggested that osteoporosis-related knowledge among women of their sample was limited, the information was not fully understood or poorly internalized. They also suggested that it is unlikely that this type of knowledge will provide a basis for decisions about life style or unhealthy habits [11]. These comments are perhaps similarly applicable to the subjects in our study. Other previous studies have obtained results consistent with this view that osteoporosis-related knowledge is often poorly integrated and internalized, and does not lead to improved health behaviours [12,16,23,24].

Our data on physical activity in the three age groups indicate that the amount of physical activity engaged in paid work currently declines with increasing age, whereas the amount of physical activity during housework appears to increase with increasing age. This trend was confirmed by multivariate analysis, which indicates that the most effective predictors of amount of physical activity at worksite or at home were age and educational level. Educational level attained likely increases the likelihood of obtaining paid work. However, recreational activities and other health-preventive behaviours such as to do isometric exercises may also determine total physical activity.

Considering all subjects together, most reported physical activity (96%) was performed in the course of daily housework, walking to work or shops, or standing at work or at home; only 4% was directly applicable to weight-bearing exercise. About 50% of women in the youngest age group did more than 2 hours of physical activity per week, but the amount of physical activity in the older women was markedly lower, and even in the youngest age group very few women performed the recommended daily minimum of 30 minutes of exercise per day [5,6]. Similar results were obtained in a previous study, which found that fewer than 50% of African-American and Hispanic women interviewed in the US do 60 minutes of physical exercise per week [16].

Very few of the women interviewed for the present study employ any of the weight-bearing physical activities that are known to be especially effective for increasing bone mass and thus perhaps reducing the risk of osteoporosis, such as isometrics [25]. Similar findings have been obtained in studies of Caucasian women in the US [11,12] and of African-American and Hispanic women residing in the US [12,16].

Independent of age and income, most women interviewed (50 – 75%) have a daily calcium intake of less than 60% of the recommended level, thus increasing the risk of osteoporosis. Similarly, in another studies of Caucasian and African-American and Hispanic women in the US [12,16], and Asian and Caucasian women in Australia [13], most of these women did not fulfil the suggested calcium intake. By contrast, in other recent study of Caucasian women in the US [11], only about 20% of women ingested less than 60% of the recommended daily calcium.

The difference between our results in El Salvador and those obtained in the study of Caucasian women in the US [11] may, at least in part, reflect differences in standard of living between these two countries. Specifically, the generally higher incomes in the US may be associated with healthier diet, higher educational level, better access to healthcare, and better public education about general health and the prevention of diseases like osteoporosis. Additionally, calcium fortified foods are more readily available in the US.

The average proportion of calcium intake as dairy products was 57% in women below the poverty line and 68% in women above the poverty line; both values are close to those reported for Caucasian women in the US [11]. These authors did not detect any significant variation with income, but note that their sample was smaller (n = 75) and more homogeneous (in terms of educational level and household income) than the sample of our study. The relationships between household income and calcium intake observed in our multivariate analyses likewise indicate that calcium intake varies with income. The other important predictor of calcium intake in this analysis was lactose intolerance, reported by 19% of the women interviewed; dairy calcium intake was likely lower in these women. As lightly lower prevalence of lactose intolerance (16%) was reported in the study of Caucasian women in the US [11].

The consumption of caffeine-containing drinks, especially coffee, has been shown to influence calcium intake, since these beverages often replace milk and milk-based beverages due to dairy products are relatively expensive in El Salvador and many Salvadorean families (42.9%) have a low purchasing power, and live below the poverty line [26]. Excessive caffeine ingestion is also reported to accelerate bone resorption [5,6], and prevents intestinal calcium absorption [9].

Calcium intake also varied with age, being significantly less among younger women. Similarly, most of interviewed Caucasian and African-American young adult women in the US did not get recommended calcium intake per day [12]. This may be because younger women tend to perceive dairy products as having a high content in animal fats, and thus tend to reject them, as it has been suggested by previous authors [19]. Auld *et al*. also suggested that Hispanic women (particularly adolescents and young women) in general tend to reject dairy products more than Anglosaxon and Asiatic women.

The key practical implications of this study relate to suggest set up of primary healthcare programs regarding osteoporosis for Salvadorean women as it has also suggested for Taiwanese women [21], and Hispanic and African-American women [16], as well as training of healthcare professionals, which in light of the present study need to pay special attention to the following aspects of osteoporosis prevention:

a) The type and frequency of physical exercise.

b) Diet-related risk factors, including inadequate intake of calcium, vitamin D, and phosphorus; also the adverse effects of drinking coffee or other low-calcium drinks instead of milk or milk-based drinks.

c) The importance of monitoring menstruation frequency, since normal circulating estrogen levels and normal menstrual cycles are important for maintaining normal bone metabolism.

In addition, it is clearly important to set up health education programs to focus on integration and internalization of knowledge about osteoporosis.

Although our results indicate that many women have modest knowledge about osteoporosis concerning risk factors and preventive behaviours, this knowledge often does not translate to appropriate changes in healthy life habits as it is shown through a weak association between total osteoporosis scores and exercise or calcium intake, so that osteoporosis knowledge is not well internalized among interviewed Salvadorean women. The lack of time to practice most adequate osteoporosis preventive exercise may accounts for the weak association between foregoing variables since majority of interviewed women perform WBPA (> 8 hours/day), but as labour activities or housework. The low calcium intake among subjects may be explained through they have a low purchasing power and do not have the habit to ingest enough dairy products, the main diet calcium source [9]. Non-dairy calcium intake including supplements was also low (< 370 mg/day), so that they did not fulfil the recommended daily calcium intake from this source either.

Finally, we would suggest that osteoporosis education campaigns should be directed at families as well as individuals, with the aim of fomenting within-family education of children about the importance of calcium in the diet, whether as dairy products or in other calcium-rich products such soya milk and oat-based drinks and desserts.

## Conclusions

It showed that Salvadorean women with secondary or higher education have significantly better knowledge about osteoporosis than women with lower educational level, regardless of age. However, this knowledge does not appear to lead to improved life style or preventive habits of osteoporosis.

The amount of weight bearing physical activity (mainly walking or standing) during paid work or housework was high in the three age groups of interviewed women (over 8 hours/day). However, very few of the women interviewed for this study did not perform any of the weight-bearing physical activities that are known to be especially effective for increasing bone mass and thus reducing the risk of osteoporosis, such as going up and down stairs, jogging, aerobics, swimming, and isometrics.

Most Salvadorean interviewed women have a daily calcium intake of 60% or less than the recommended level depending of lactose intolerance, household income, age, and coffee consumption. The former variables directly affect both calcium source diversity and intake frequency. Age would influence dairy calcium intake, since younger women tend to perceive dairy products as having a high animal fat content, and thus reject them. The coffee consumption would affect calcium intake, since this beverage often replace milk. Therefore, all these factors together may be increasing the risk to develop osteoporosis among Salvadorean women.

## List of abbreviations

WBPA: Weight-bearing physical activity

ANOVA: Analysis of Variance

SEM: Standard error of mean

## Competing interests

None declared.

## Authors' contributions

RHR performed the design of the study, coordinated the study, performed data analysis, and drafted the paper. SMG participed in the design of the study and provided input into the paper.

## Pre-publication history

The pre-publication history for this paper can be accessed here:



## Figures and Tables

**Table 1:** Knowledge dimensions about osteoporosis, specific questions, and corresponding scores get through concept integration.

Knowledge dimensions about osteoporosis and corresponding questions	None related concept, sub-scores	One related concept, sub-scores	Up to two related concepts, sub-scores	≥ Three related concepts, sub-scores
**Concept related ***What you can tell me about osteoporosis?*	0	2	3	5
**Symptom related ***What changes do you note in your body if you have osteoporosis?*	0	2	3	5
**Affected bones **If one bone or more was mentioned when previous question was done.	0	2	3	5
**Sex-related factor ***Are women and men equally prone to osteoporosis, or is it more frequent in one of the sexes?*	0	2	---	---
**Risk-factor related ***What factors increase the risk of suffering osteoporosis?*	0	2	3	5
**Prevention behavior related ***How can you reduce the risk of suffering osteoporosis?*	0	2	3	5
**Diagnosis related ***How can osteoporosis be detected?*	0	2	3	5
**Actual treatment related ***How can osteoporosis be treated?*	0	2	3	5
**Osteoporosis information source ***Where did you get the information about osteoporosis?*	0	2	3	5
Maximum score (Σ)				42

**Table 2:** Descriptive data from a convenience sample, according to women's age-group.

Characteristics	25–35 years (n = 73)	36–49 years (n = 74)	over 49 years (n = 50)
*Age*-Mean (SEM)	29.8 (0.4)	42.4 (0.5)	64.5 (1.5)
*Educational level *(%)			
None	3 (4.1)	15 (20.3)	14 (28.0)
Primary	23 (31.5)	27 (36.5)	25 (50.0)
Secondary	17 (23.3)	9 (12.1)	4 (8.0)
Higher	30 (41.1)	23 (31.1)	7 (14.0)
*Household income *(%)			
Above the poverty line	28 (38.4)	28 (37.8)	13 (26.0)
Below the poverty line	45 (61.6)	46 (62.2)	37 (74.0)
*Fecundity *(%)			
Children per woman^†^-Mean (SEM)	2.4 (0.2)	2.7 (0.1)	6.0 (0.5)
Nulliparity	16 (21.9)	5 (6.8)	5 (10.0)
Parity	57 (78.1)	69 (93.2)	45 (90.0)
*Family history of osteoporosis *(%)			
Yes	40 (54.8)	33 (44.6)	32 (64.0)
No	33 (45.2)	41 (55.4)	18 (36.0)
*Menopause among women *(Irrespective of age group).			
Mean age (years)	42.3 ± 0.8		
Median age (years)	44.0		
Range of age (years)	27.0 – 53.0		
*Menopause before age 45 years *(%)	25–35 years (n = 73)	36–49 years (n = 74)	Over 49 years (n = 50)
Yes	4 (5.5)	18 (24.3)	18 (36.0)
No	69 (94.5)	56 (75.7)	30 (64.0)
*Use of aluminum antiacids *(%)			
Users	16 (21.9)	17 (23.0)	13 (26.0)
Non-users	57 (78.1)	57 (77.0)	37 (74.0)
*Lactose intolerance *(%)			
Yes	15 (20.5)	14 (18.9)	9 (18.0)
No	58 (79.5)	60 (81.1)	41 (82.0)
*Coffee consumption *(%)			
Yes	51 (69.9)	62 (83.8)	39 (78.0)
No	22 (30.1)	12 (16.2)	11 (22.0)
Cups a day^‡^-Mean (SEM)	1.8 (0.1)	2.1 (0.1)	1.9 (0.1)

^† ^Fecundity rate is only based on data of parity per each age group.

^‡ ^Consumption rate is only based on coffee-consumer data per each age group.

**Table 3:** Osteoporosis knowledge scores according knowledge dimensions and women's age-groups.

Knowledge dimensions about osteoporosis (expressed as scores).^†^	25–35 years (n = 73)^a^	36–49 years (n = 74)^a^	Over 49 years (n = 50)^a^
*Concept related*	3 (5)	3 (5)	3 (5)
*Symptom related*	2 (3)	3 (3)	3 (3)
*Affected bone related*	0 (3)	2 (2)	0 (2)
*Sex related*	2 (2)	2 (2)	2 (2)
*Risk factor related*	3 (5)	3 (5)	3 (5)
*Prevention behavior related*	3 (5)	3 (5)	3 (5)
*Diagnosis related*	0 (3)	0 (3)	0 (3)
*Actual treatment related*	0 (0)	0 (0)	0 (0)
*Osteoporosis information source*	1 (2)	1 (2)	1 (2)
Σ	14 (28)	17 (27)	15 (27)

^† ^All values are median and 75^th ^percentile (in brackets) of reached scores in each knowledge areas, per each age group.

^a ^Comparisons among scores of knowledge specific area per each age group were not significantly different (p > 0.05, Kruskal-Wallis ANOVA on ranks).
